# Supplementary material for: Diversity spectrum analysis identifies mutation-specific effects of cancer driver genes
Source: Commun Biol. 2020 Jan 7;3:6. doi: 10.1038/s42003-019-0736-4 (PMC6946677; doi:10.1038/s42003-019-0736-4)
Supplement: Supplementary file 1 — Supplementary Information [file 42003_2019_736_MOESM1_ESM.pdf]

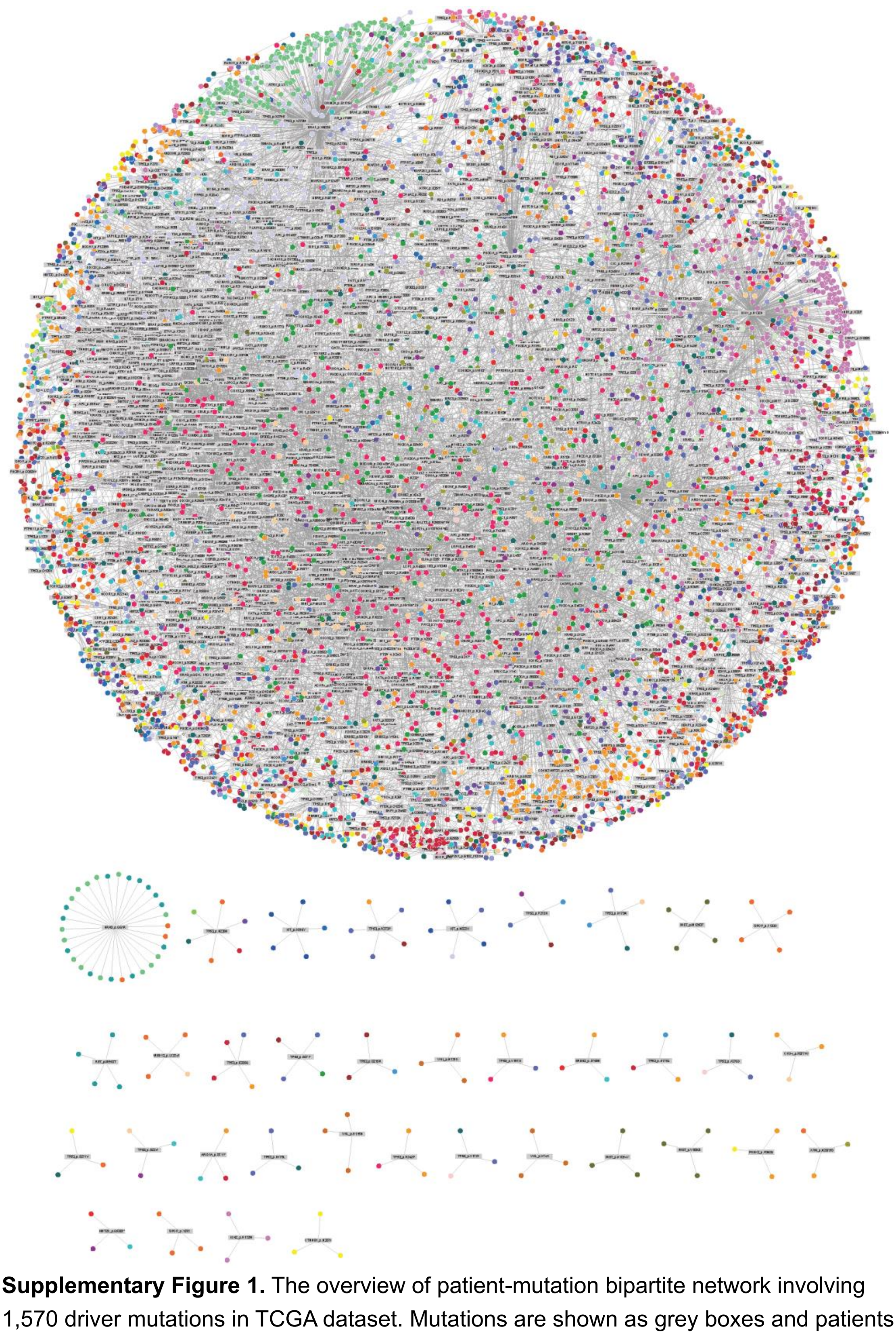

Supplementary Figure 1. The overview of patient-mutation bipartite network involving 1,570 driver mutations in TCGA dataset. Mutations are shown as grey boxes and patients are shown as colored dots. Different colors mark different cancer types. The details of this network can be found in Supplementary Data 15.

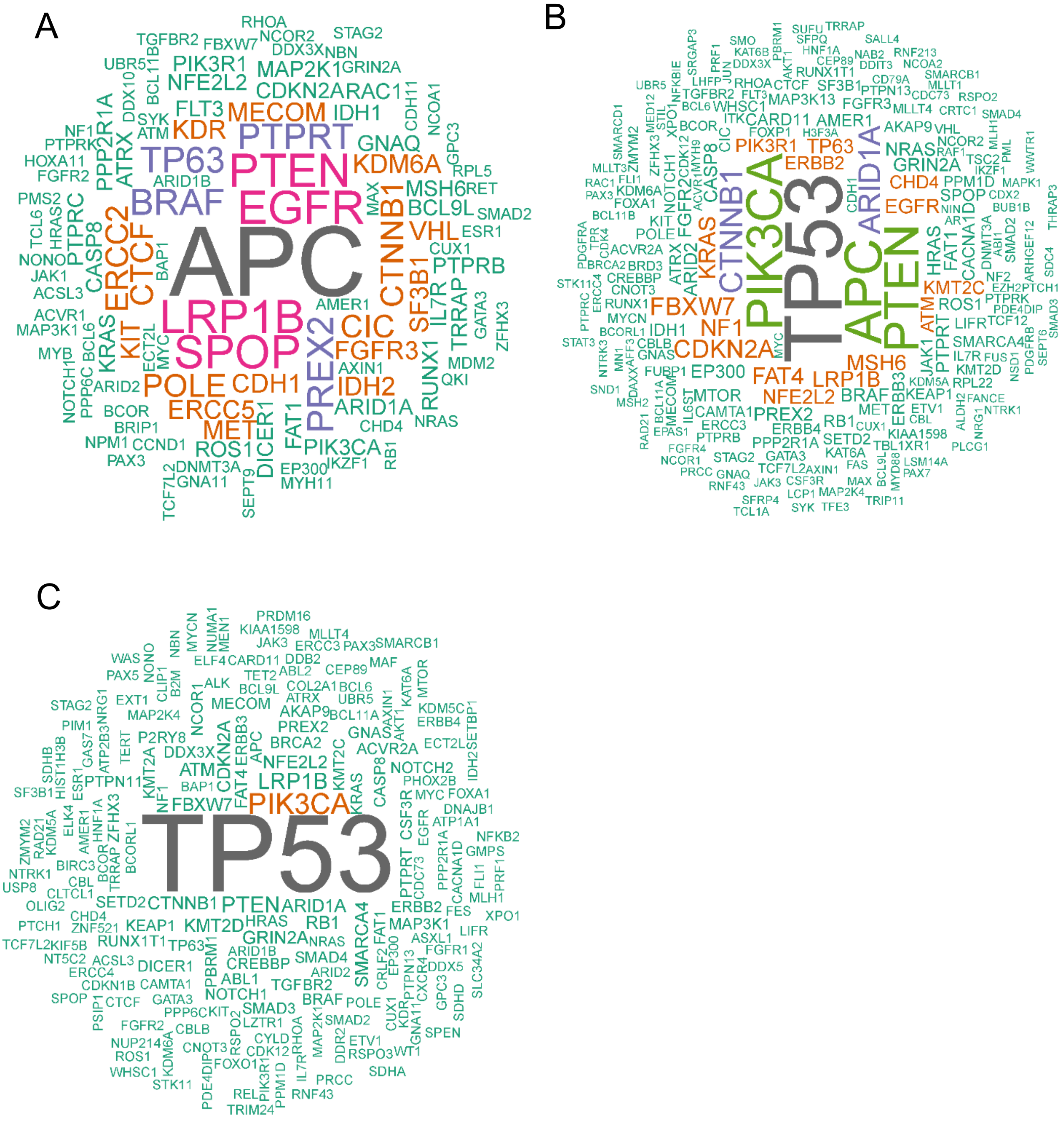

Supplementary Figure 2. The word clouds show the distribution of genes in SPM (A), RSM (B) and PCM (C) categories.

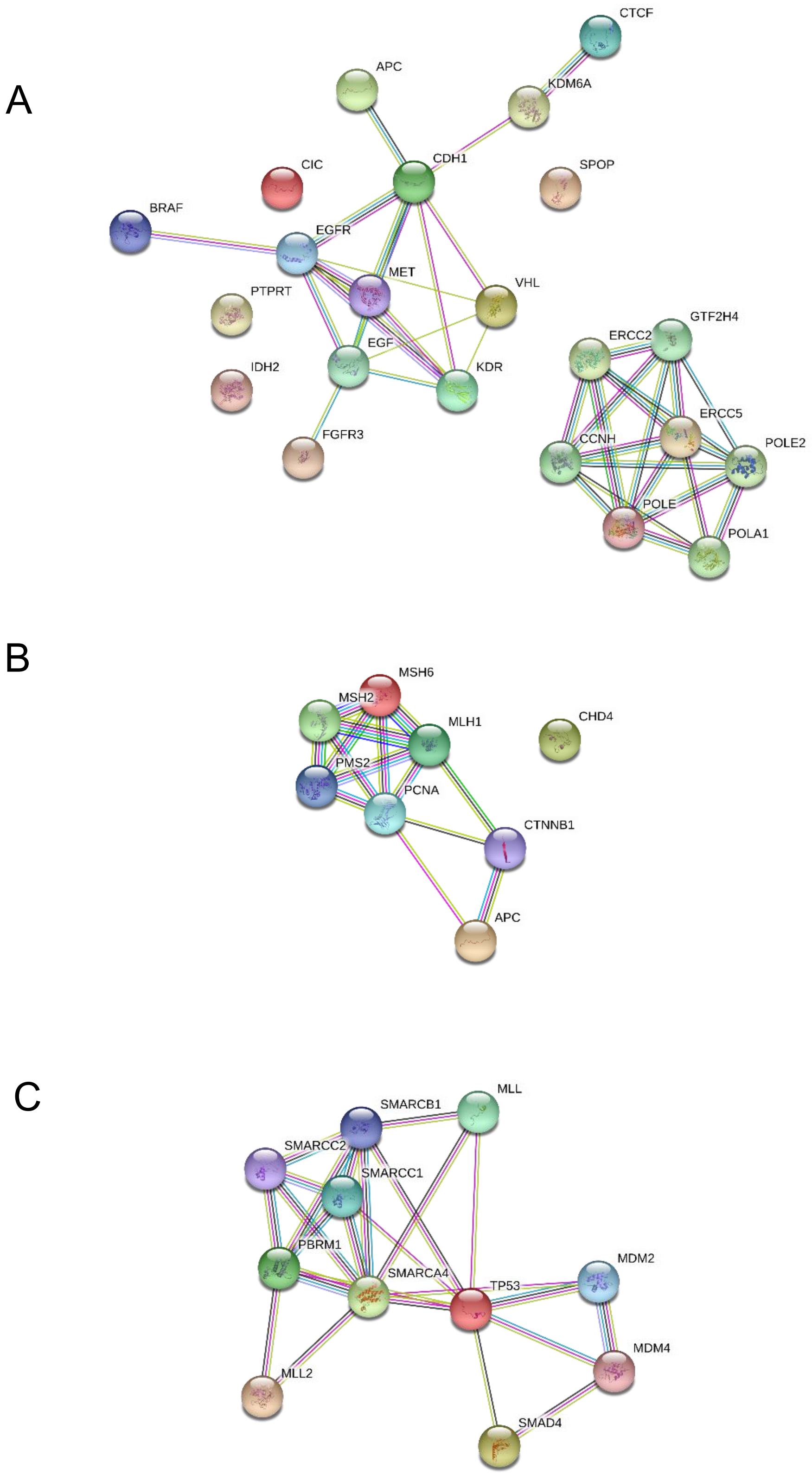

Supplementary Figure 3. The extended gene networks using driver genes enriched with SPMs (A), RSMs (B) and PCMs (C), respectively. These networks are constructed using STRING database.

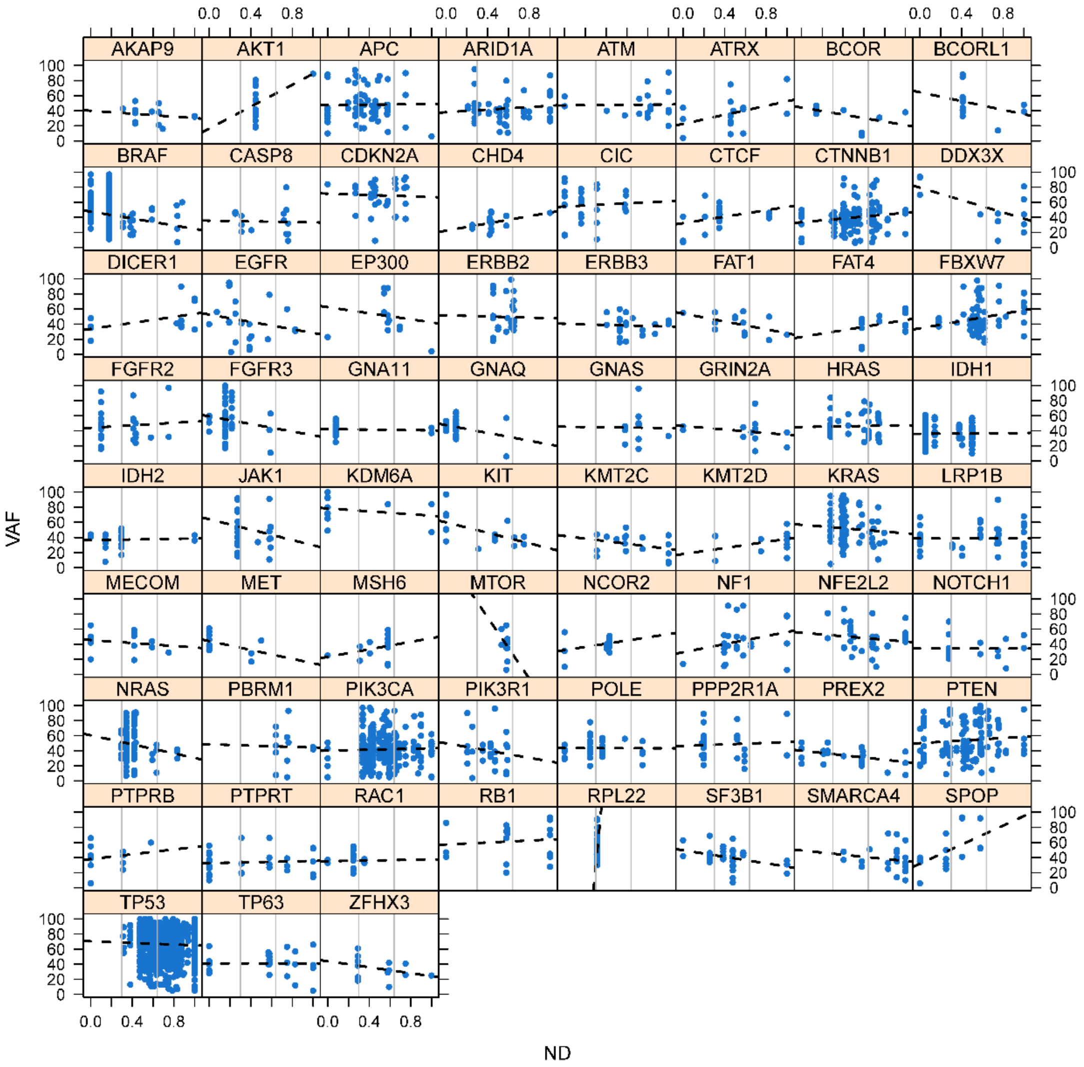

Supplementary Figure 4. The correlation of network diversity and VAF.

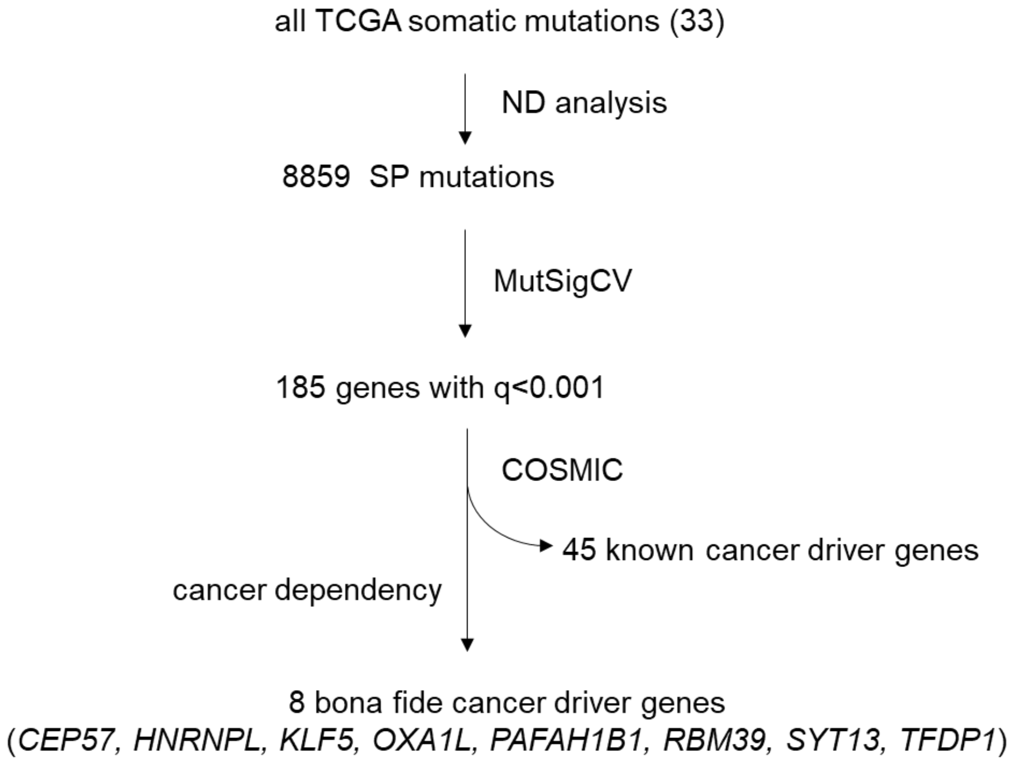

Supplementary Figure 5. The workflow of discovery of new cancer driver genes. ND represents network diversity here.

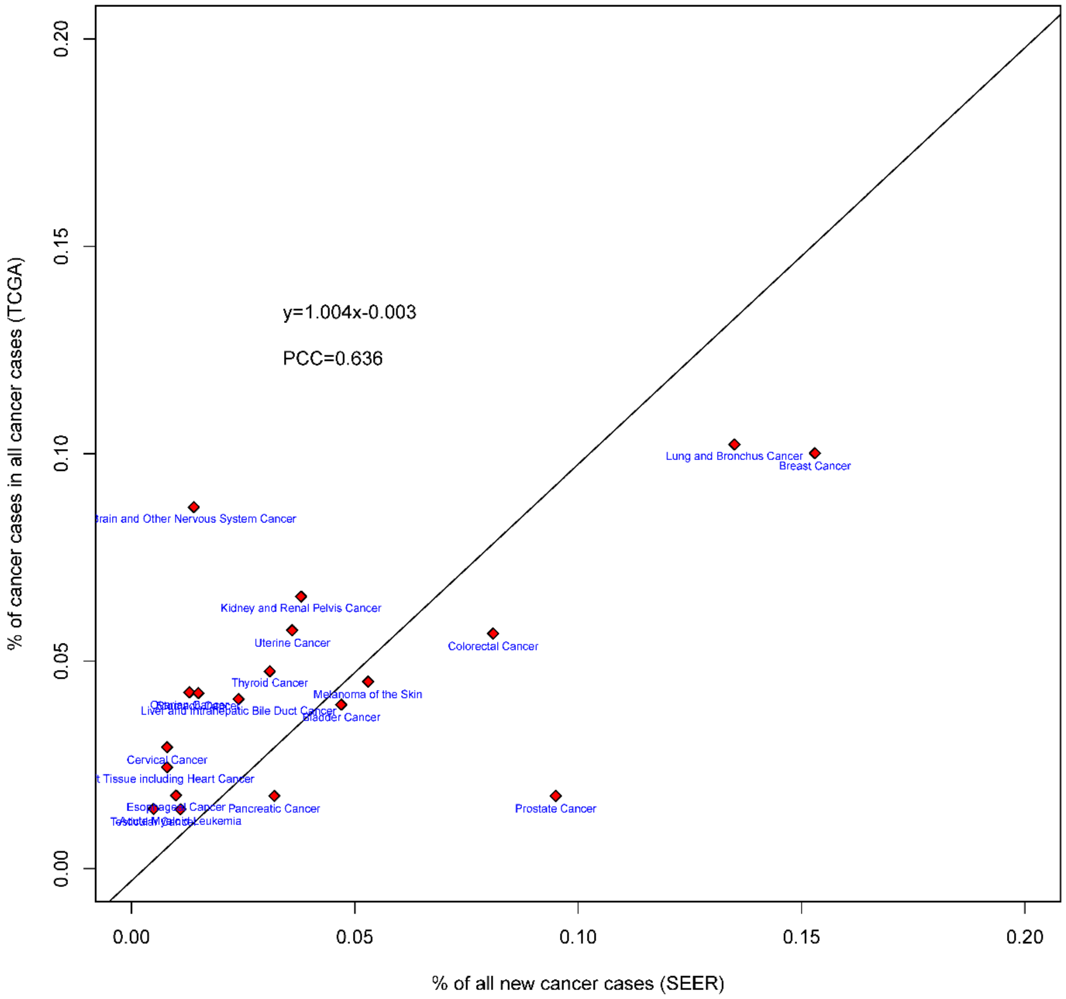

Supplementary Figure 6. The correlation of epidemiology data in the Surveillance, Epidemiology, and End Results (SEER) database and sample size composition in TCGA.

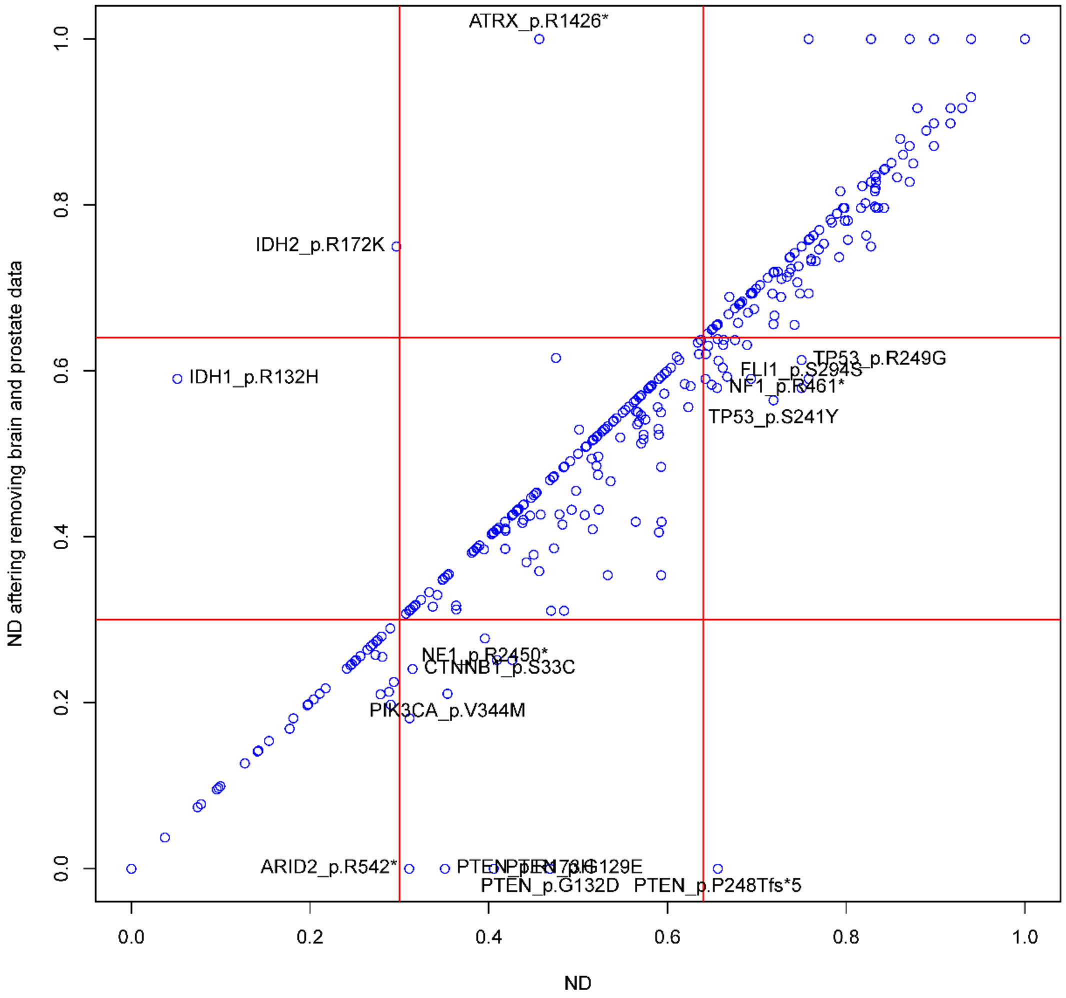

Supplementary Figure 7. Recomputed network diversity values after samples from brain and prostate cancer samples was removed. The two cancer types were outliers in sample size-epidemiology correlation analysis.

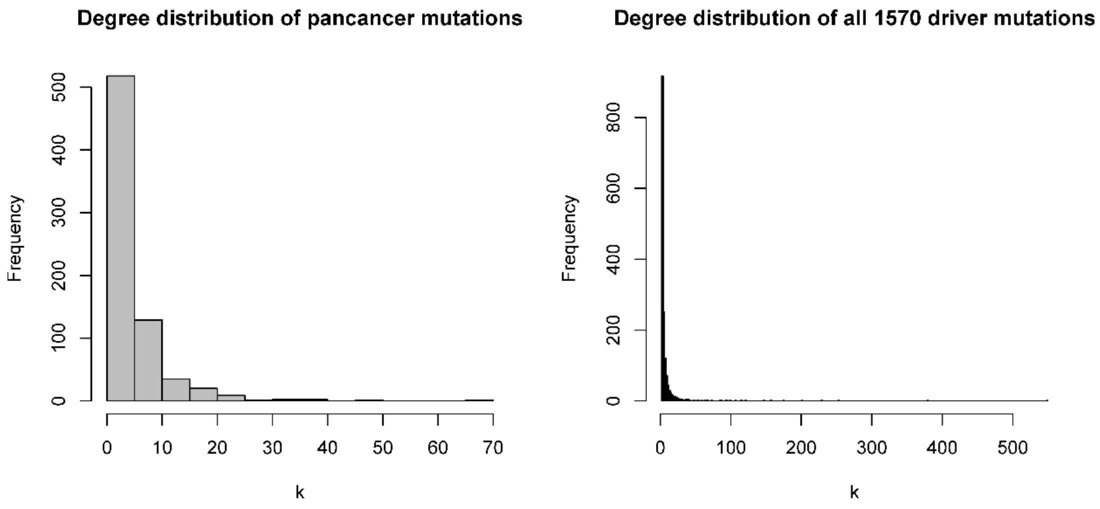

Supplementary Figure 8. The degree distribution of PCMs and all driver mutations.

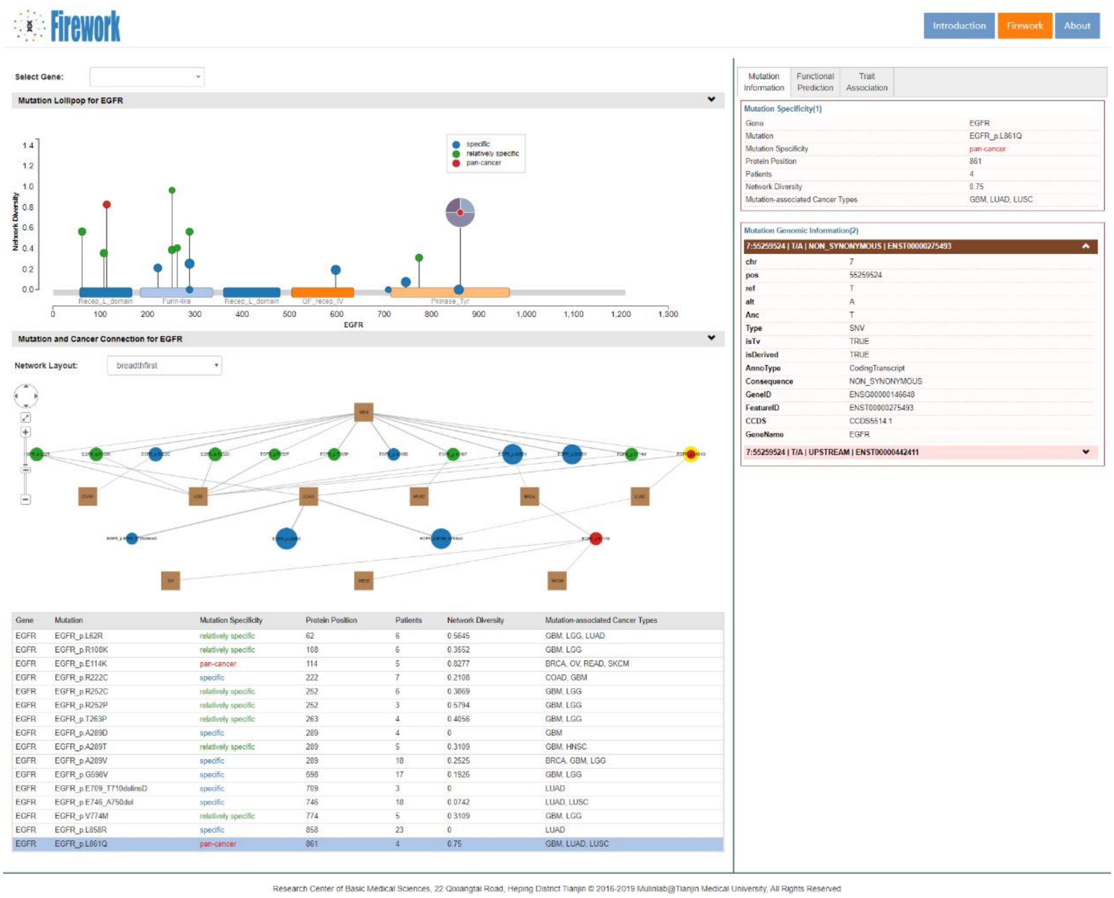

Supplementary Figure 9. The web portal named Firework (http://milinalb.org/firework/firework.html) can be used to query and visualize cancer diversity of driver mutations.

Supplementary Discussion

There are limitations in this study. First, the coverage of driver mutations analyzed in this study is moderate, and the majority of our observations are only based on approximately 1,500 driver mutations. This low number could be attributed to the fact that many driver mutations are rarely observed in current cancer genomics cohorts, such as the TCGA project, which limits the measurement of their cancer diversity. It is possible to improve the coverage if more data sources are included; however, factors such as the standard of patient enrollment and data processing could confound subsequent analyses. As we have shown, the cancer specificity driver mutations are influenced by the stage of tumors. The second limitation of this study is that no proper method was used to evaluate the uncertainty of each NDnetwork diversity value. A nonparametric permutation test is also not suitable because the random operation will inevitably lead to the high entropy of the network; hence, a sophisticated algorithm is needed to solve this dilemma. Third, our approach requires that a mutation is recurrent in cancers, otherwise, the computing of network diversity would be impossible or the value would be unreliable. Considering that gain-of-function mutations tend to occur at a specific position and the loss-of-function mutations occur relatively randomly on a gene, the recurrence rate of gain-of-function mutations would be higher and more of them would satisfy the requirement of our approach but some loss-of-function mutations would be missed. We think that a possible solution for this shortcoming would be clustering some very similar loss-of-function mutations before performing cancer diversity analysis, which can improve the recurrence rate of them. Finally, to perform the mutation cancer diversity analysis more comprehensively, abundant cancer genome data across as many cancer types as possible will be needed, which is impossible in the era of focusing on a single cancer type. Because whole genome or exome sequencing is becoming standard in clinical oncology, the accumulation of gene mutations in more cancer types and more patients will alleviate the above problems in our method.

Our study tries to characterize the distribution of cancer driver mutations and relate it to the functional difference of these drivers. Ideally, the cancer type composition of samples should consist with the distribution of cancer epidemiology. The correlation

between our original sample size of each cancer type in the TCGA and the cancer epidemiology data in the Surveillance, Epidemiology, and End Results (SEER) database (https://seer.cancer.gov/statfacts/) is high (PCC=0.636, Supplementary Figure 6), indicating the pancancer analysis based on our samples is representative. To test the robustness of our results, we also re-computed the network diversity values of cancer driver mutations after removing brain and prostate cancer datasets, since they were outliers in the sample size-epidemiology correlation analysis. We only found 4% of retained mutations (56/1438) were misclassified in cancer diversity spectrum (Supplementary Figure 7). In addition, considering the network diversity value may be inflated by small sample size, we analyzed the distribution of PCMs as a function of k (degree in patient-mutation network or the number of associated patients) and compared it with that of all 1,570 driver mutations (Supplementary Figure 8). We found no significant difference between them, and there is no enrichment of rare mutations (defined as k = 3) in PCMs (269/718 vs 626/1570, p-value=0.959, hypergeometric test).

Patients with hereditary cancer predisposition syndromes suffer from high cancer risk since they carry germline cancer driver mutations. Even these mutations exist in all tissues and cells of the whole body, the risks of tumorigenesis vary with tissue types and mutated genes [1], which could provide an orthogonal validation of the cancer mutation specificity learned from TCGA somatic mutation data using network diversity measurement.

To this end, we collected germline-derived cancer predisposition information for driver genes that were marginally enriched in each mutation category (Fisher's exact test, p-value<0.05, without multiple hypothesis correction). As expected, the enriched categories of driver genes consistently depict the tissue specificities of germline-derived cancer predisposition syndromes (Supplementary Data 3). For instance, *APC*, *ERCC2*, *KDR*, *VHL* and are enriched in the SPM mutation category (SPM genes), and their germline variants will lead to highly tissue-specific hereditary cancer predisposition syndromes, including familial adenomatous polyposis (colorectum), xeroderma pigmentosum (skin), hemangioma (blood vessel), Von Hippel-Lindau syndrome (kidney) and hereditary diffuse gastric cancer (stomach), respectively [1]. The DNA mismatch repair

gene *MSH6* is enriched in the RSM mutation category (RSM gene), and its germline variants could cause Lynch syndrome in which patients have a high risk of colon cancer as well as other cancer types, such as gastrointestinal cancer, but with a relatively lower risk [2]. Finally, germline variants of *TP53*, which are enriched in the PCM mutation category (PCM gene), will lead to the risk improvement of almost all cancer types, which is also known as Li-Fraumeni syndrome [3].

Reference

1. Schneider G, Schmidt-Supprian M, Rad R, Saur D. Tissue-specific tumorigenesis: context matters. Nature Reviews Cancer 17, 239 (2017).
2. Møller P, et al. Cancer risk and survival in path_MMR carriers by gene and gender up to 75 years of age: a report from the Prospective Lynch Syndrome Database. Gut 67, 1306-1316 (2018).
3. McBride KA, et al. Li-Fraumeni syndrome: cancer risk assessment and clinical management. Nature Reviews Clinical Oncology 11, 260 (2014).
