# Supplementary material for: Diversity spectrum analysis identifies mutation-specific effects of cancer driver genes
Source: Commun Biol. 2020 Jan 7;3:6. doi: 10.1038/s42003-019-0736-4 (PMC6946677; doi:10.1038/s42003-019-0736-4)
Supplement: Supplementary file 2 — Description of additional supplementary items [file 42003_2019_736_MOESM2_ESM.docx]

Other materials for this manuscript are included in following:

1. Supplementary Data 1 to 14 (in Excel format)
   1. **Supplementary Data 1.** Selected driver mutations
   2. **Supplementary Data 2.** Network diversity of 1,570 TCGA driver mutations
   3. **Supplementary Data 3.** Driver genes and hereditary cancer predisposition
   4. **Supplementary Data 4**. Functional enrichment analysis of extended SPM enriched gene network
   5. **Supplementary Data 5.** Functional enrichment analysis of extended RSM enriched gene network
   6. **Supplementary Data 6.** Functional enrichment analysis of extended PCM enriched gene network
   7. **Supplementary Data 7.** Cancer diversity and drug sensitivity
   8. **Supplementary Data 8**. Evidence levels of druggable mutations in OncoKB
   9. **Supplementary Data 9.** Network diversity of shared driver mutations between TCGA and MSK-IMPACT
   10. **Supplementary Data 10.** Co-mutation pairs
   11. **Supplementary Data 11.** Network diversity analysis is extended to all recurrent TCGA mutations
   12. **Supplementary Data 12.** The 184 genes that are significantly enriched with SPMs
   13. **Supplementary Data 13.** Abbreviations and full names of 33 TCGA cancer types
   14. **Supplementary Data 14.** The accession codes of TCGA data
2. **Supplementary Data 15**. (in zip format) Patient-mutation network.cys. The network files of patient-mutation networks in TCGA and MSK-IMPACT (Cytoscape file)
