# Supplementary material for: Diversity spectrum analysis identifies mutation-specific effects of cancer driver genes
Source: Commun Biol. 2020 Jan 7;3:6. doi: 10.1038/s42003-019-0736-4 (PMC6946677; doi:10.1038/s42003-019-0736-4)
Supplement: Supplementary file 18 — Reporting Summary [file 42003_2019_736_MOESM18_ESM.pdf]

nature research

Corresponding author(s): Mulin Jun Li

Last updated by author(s): Dec 5, 2019

Reporting Summary

Nature Research wishes to improve the reproducibility of the work that we publish. This form provides structure for consistency and transparency in reporting. For further information on Nature Research policies, see Authors & Referees and the Editorial Policy Checklist.

Statistics

For all statistical analyses, confirm that the following items are present in the figure legend, table legend, main text, or Methods section.

n/a	Confirmed	
☐	☒	The exact sample size (*n*) for each experimental group/condition, given as a discrete number and unit of measurement
☐	☒	A statement on whether measurements were taken from distinct samples or whether the same sample was measured repeatedly
☐	☒	The statistical test(s) used AND whether they are one- or two-sided<br>*Only common tests should be described solely by name; describe more complex techniques in the Methods section.*
☒	☐	A description of all covariates tested
☐	☒	A description of any assumptions or corrections, such as tests of normality and adjustment for multiple comparisons
☐	☒	A full description of the statistical parameters including central tendency (e.g. means) or other basic estimates (e.g. regression coefficient) AND variation (e.g. standard deviation) or associated estimates of uncertainty (e.g. confidence intervals)
☐	☒	For null hypothesis testing, the test statistic (e.g. *F*, *t*, *r*) with confidence intervals, effect sizes, degrees of freedom and *P* value noted<br>*Give P values as exact values whenever suitable.*
☒	☐	For Bayesian analysis, information on the choice of priors and Markov chain Monte Carlo settings
☒	☐	For hierarchical and complex designs, identification of the appropriate level for tests and full reporting of outcomes
☒	☐	Estimates of effect sizes (e.g. Cohen's *d*, Pearson's *r*), indicating how they were calculated

Our web collection on statistics for biologists contains articles on many of the points above.

Software and code

Policy information about availability of computer code

Data collection	Publicly available data used in this study were downloaded from corresponding websites using Google web browser Chrome or wget tool on Linux.TCGA Mutation Annotation Format files for 33 cancer types were downloaded from the Genomic Data Commons (GDC) data portal. Cancer genes and information for their roles in cancer were downloaded from COSMIC (v81). Scores of M-CAP were downloaded from its website. IDWAS data matrix was obtained from the supplemental materials in the study by Geeleher et al. Gene-drug interaction data were downloaded from DGIdb. Clinical drug recommendation information was downloaded from the OncoKB. Tumor purity data estimated by InfiniumPurify were downloaded from corresponding supplemental materials. Level 3 CNV data generated by Affymetrix SNP 6.0 array were downloaded from the GDC data portal. Targeted sequencing data of advanced tumors generated by MSK-IMPACT were downloaded from the cBioPortal. The PDB file was downloaded from the Protein Data Bank.
Data analysis	Python language (v2.7) were used for most analysis. Network analysis were conducted with Python package NetworkX. Cytoscape (> v3.0) were used for network visualization. R language (v3.4) were used for statistical analysis. The MutSigCV module in GenePattern public server was used to search for driver genes. Pathway enrichment analyses were performed with STRING webserver. The impact of the expression of these eight genes expression on patient survival was analyzed by querying the Human Pathology Atlas. The protein 3D structure was visualized with Pymol.

For manuscripts utilizing custom algorithms or software that are central to the research but not yet described in published literature, software must be made available to editors/reviewers. We strongly encourage code deposition in a community repository (e.g. GitHub). See the Nature Research guidelines for submitting code & software for further information.

Data

Policy information about availability of data

All manuscripts must include a data availability statement. This statement should provide the following information, where applicable:

- Accession codes, unique identifiers, or web links for publicly available datasets
- A list of figures that have associated raw data
- A description of any restrictions on data availability

We only used publicly available data. The data sources have been described in Methods. Cancer diversity of driver mutations can be visualized at http://mulinlab.org/firework and related data can be found in supplementary data. Computer code is available on request.

Field-specific reporting

Please select the one below that is the best fit for your research. If you are not sure, read the appropriate sections before making your selection.

☒ Life sciences ☐ Behavioural & social sciences ☐ Ecological, evolutionary & environmental sciences

For a reference copy of the document with all sections, see nature.com/documents/nr-reporting-summary-flat.pdf

Life sciences study design

All studies must disclose on these points even when the disclosure is negative.

Sample size	We used all samples we could obtain from public data source.
Data exclusions	See Methods.
Replication	No experiments were performed.
Randomization	We only used publicly available data and randomization was not relevant.
Blinding	We only used publicly available data and blinding was not relevant.

Reporting for specific materials, systems and methods

We require information from authors about some types of materials, experimental systems and methods used in many studies. Here, indicate whether each material, system or method listed is relevant to your study. If you are not sure if a list item applies to your research, read the appropriate section before selecting a response.

Materials & experimental systems

n/a	Involved in the study	
☒	☐	Antibodies
☒	☐	Eukaryotic cell lines
☒	☐	Palaeontology
☒	☐	Animals and other organisms
☒	☐	Human research participants
☒	☐	Clinical data

Methods

n/a	Involved in the study	
☒	☐	ChIP-seq
☒	☐	Flow cytometry
☒	☐	MRI-based neuroimaging
